# Prenatal diagnosis and postnatal follow-up of 15 fetuses with 16p13.11 microduplication syndrome

**DOI:** 10.3389/fgene.2024.1486974

**Published:** 2024-10-15

**Authors:** Yan Zhao, Lina Song, Shuxia Zhang, Fei Hou, Shan Shan, Hua Jin

**Affiliations:** ^1^ Antenatal Diagnostic Center, Jinan Maternity and Child Care Hospital Affiliated Shandong First Medical University (Jinan Maternity and Child Care Hospital), Jinan, Shandong, China; ^2^ Department of Obstetrics and Gynecology, Qixia City People’s Hospital, Yantai, Shandong, China; ^3^ Medical Research Center, Jinan Maternity and Child Care Hospital Affiliated Shandong First Medical University (Jinan Maternity and Child Care Hospital), Jinan, China

**Keywords:** 16p13.11, SNP-array, microduplication, CNVs, prenatal diagnosis

## Abstract

**Background:**

The clinical phenotypes of 16p13.11 microduplication syndrome have been extensively reported in previous studies, mostly about adults and children, with limited information available on fetal cases. This study aims to explore the genotype-phenotype correlation of fetuses with 16p13.11 microduplication syndrome and analyze the characteristics of prenatal diagnosis indications and provide clinical information for prenatal and postnatal genetic counseling.

**Methods:**

We conducted a retrospective analysis of 3,451 pregnant women who underwent invasive prenatal diagnosis for SNP array between January 2018 and December 2022 at the Jinan Maternal and Child Health Hospital. Descriptive statistical analysis was performed on the prenatal diagnosis indications, pedigree analysis, pregnancy outcomes and postnatal follow-up of 15 fetuses with 16p13.11 microduplication syndrome.

**Results:**

SNP array revealed that 15 fetuses had duplications in the 16p13.11 region with varying prenatal diagnosis indications. Among the cases, 6/15 exhibited ultrasound abnormalities, 5/15 had abnormal chromosomal copy number variations as indicated by non-invasive prenatal testing (NIPT), one case involved advanced maternal age, and 3/15 had other abnormalities. 16p13.11 microduplication syndrome was closely related to ultrasound abnormalities, especially structural abnormalities and soft marker anomalies (abnormal ultrasonic soft indicators), while the indication of NIPT could improve the detection rate of copy number variations (CNVs) in this region. Only 7/15 fetuses underwent pedigree verification, with one case of *de novo* 16p13.11 microduplication, and the others inherited from one parent. Pregnancy was terminated in 2/15 cases and the outcome of one case is unknown due to loss to follow-up. Among the remaining cases, only one case exhibited a ventricular septal defect, while another presented with omphalocele. No other obvious abnormalities were reported postnatally.

**Conclusion:**

The prenatal phenotypes of fetuses with 16p13.11 microduplication were highly associated with ultrasound abnormalities but lacked specificity. Comprehensive genetic tracing, outcome analysis, and follow-up are essential for providing accurate prenatal and postnatal genetic counseling.

## 1 Introduction

16p13.11 microduplication is a rare genetic disorder with incomplete penetrance and variable expressivity, associated with a range of neurodevelopmental and congenital disorders such as developmental delay, intellectual disability, language deficits, behavioral abnormalities, dysmorphic facial features, attention deficit hyperactivity disorder (ADHD), autism, and cardiovascular anomalies (Hamad et al., 2023). The 16p13.11 microduplication is a susceptibility factor for various neurodevelopmental disorders, though its molecular mechanisms, and candidate genes remain unclear (Watson et al., 2014). Chromosome 16 is rich in segmental duplications (SDs), also known as low copy repeats (LCRs), which account for more than 10% of its euchromatic content (Loftus et al., 1999; Martin et al., 2004; Ramalingam et al., 2011). These chromosome rearrangements are an important cause leading to neurodevelopmental disorders (Ingason et al., 2011).

The pathogenicity of 16p13.11 microdeletion has been well-established, yet the clinical significance of 16p13.11 microduplication remains controversial (Allach El Khattabi et al., 2020). Ullmann (Ullmann et al., 2007) firstly reported the pathogenicity of 16p13.1 microduplication, primarily linking it to intellectual disability and autism spectrum disorders. While Hannes (Hannes et al., 2009) identified recurrent reciprocal duplication of 16p13.1 was a common population variant (5/1682, 0.29%) suggesting that it may be a rare benign variation. In recent years, numerous copy number variations (CNVs) have been detected resulting from the widespread clinical application of chromosome microarray analysis (CMA). It is now broadly accepted that 16p13.1 microduplication may be associated with cognitive impairment, developmental delay, behavioral abnormalities, delayed language development, schizophrenia, and a variety of congenital anomalies, including cardiovascular abnormalities, skeletal abnormalities, aortic disease, renal and urinary system malformations (Kirov et al., 2009; Gumuslu et al., 2015; Erhart et al., 2018; Calderoni et al., 2020). However, individuals with normal phenotypes and unaffected relatives have also been identified, that complicating the interpretation of clinical significance of 16p13.11 microduplications in the context of prenatal genetic counseling, particularly.

Notably, most previous literatures on 16p13.11 microduplications have focused on adults and children, but limited data available on prenatal fetal phenotypes. In this study, 15 fetuses with recurrent 16p13.11 microduplication were identified using single-nucleotide polymorphism microarray (SNP array) technology. We retrospectively analyzed the prenatal diagnosis indications, parental origin, pregnancy outcome and follow-up results of fetus with 16p13.11 microduplication syndrome to further explore the genotype-phenotype correlations and elucidate the underlying genetic mechanisms of 16p13.11 microduplication syndrome.

## 2 Materials and methods

### 2.1 Subject

This retrospective analysis was conducted on data from 3,451 pregnant women who underwent invasive prenatal diagnosis by SNP array testing at the Prenatal Diagnosis Center of Jinan Maternal and Child Health Hospital between January 2018 and December 2022. Only 15 cases with 16p13.11 microduplication and no other pathogenic CNVs were included in this study. The mean age of the pregnant women was 30.5 years (range: 21–42 years), and the mean gestational age was 21 weeks (range: 18–27 weeks). All participants underwent transabdominal amniocentesis after signing informed consent and received genetic counseling from professional genetic doctor. This study was approved by the Medical Ethics Committee of Jinan Maternal and Child Health Hospital.

### 2.2 DNA extraction

Amniotic fluid samples from the pregnant women or peripheral blood from the couples were collected between 18 and 24 weeks of gestation following informed consent. DNA extraction was performed using the Tiangen (Beijing) Whole Blood/Tissue Genomic DNA Extraction Kit (Catalog No: DP304) and adhering strictly to the kit’s protocol. Genomic DNA was quantified using the NanoDrop 2000 spectrophotometer (Thermo Scientific) and stored at −20°C.

### 2.3 SNP array

Whole-genome DNA were processed through digestion, ligation, polymerase chain reaction (PCR), purification, fragmentation, labeling and hybridization steps using the Affymetrix CytoScan750K array. Data calculation and interpretation were carried out using Chromosome Analysis Suite (ChAS) version 4.0.

### 2.4 Database

The pathogenicity of the identified CNVs was further evaluated based on relevant databases. The following databases were mainly referred to: International public Database of Genomic Variants (DGV) (http://dgv.tcag.ca/dgv/app/home), DECIPHER Pathological Variation Database (http://decipher.sanger.ac.uk/browser), the International Standards for Cytogenomic Arrays Consortium (ISCA) (https://www.iscaconsortium.org/), the Online Mendelian Inheritance in Man (OMIM) (http://www.omim.org) and the Clinical Genome Resource (GlinGen) (http://www.clinicalgenome.org). Additionally, GENE, PubMed, and other relevant sources were consulted for further insights.

### 2.5 Pathogenicity assessment

The variation classification of CNVs pathogenicity followed the guidelines set forth by the American College of Medical Genetics and Genomics (ACMG, 2019). The CNVs were scored according to the following criteria:

①: Pathogenic: 0.99 points or higher, ②Likely Pathogenic: 0.90 to 0.98 points, ③Uncertain Significance: −0.89 to 0.89 points, ④Likely Benign: −0.90 to −0.98 points, ⑤Benign: −0.99 points or lower.

### 2.6 Follow-up

All fetuses underwent regular prenatal examinations with dynamic monitoring of fetal growth and development via ultrasound. Pregnancy outcomes and neonatal health status were obtained through telephone follow-up. For live births, regular telephone follow-ups were conducted to record the child’s growth, development milestones and language development. The most recent follow-up was completed in May 2023.

## 3 Results

### 3.1 SNP-array results for the fetus

**FIGURE 1:**
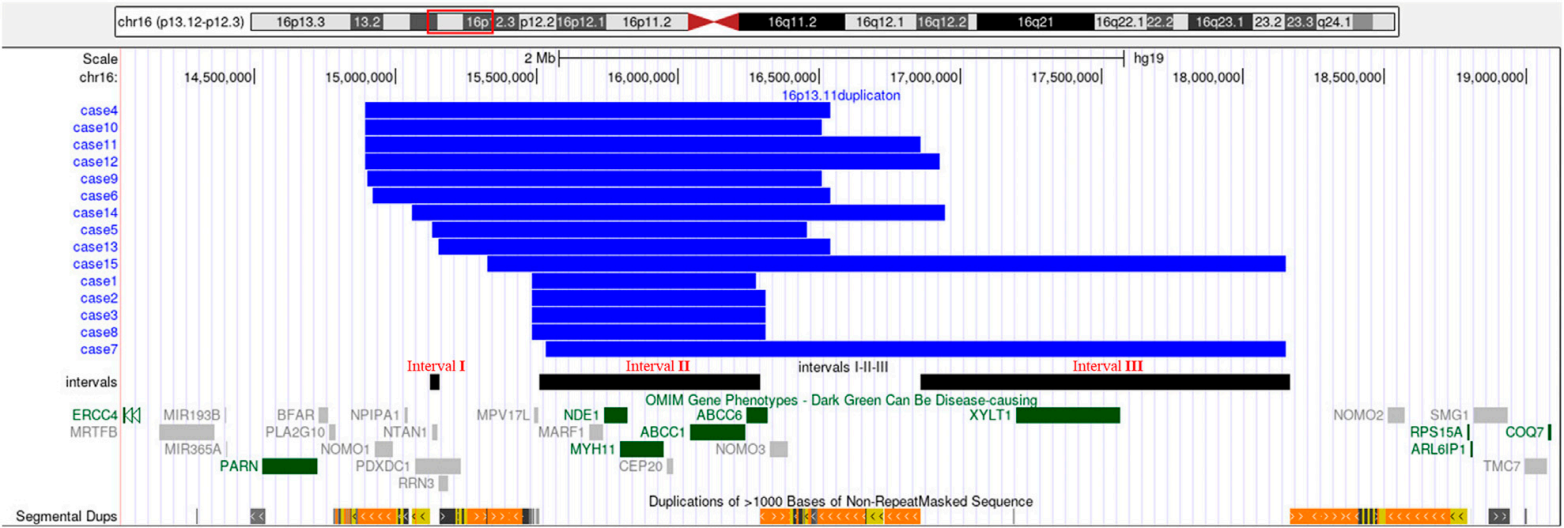
16p13.11 microduplication detected using SNP-array. SNP-array revealed 16p13.11 microduplication in fetus case1∼case15. Refer to the intervals I, II and III marked in the figure to determine the location of the 16p13.11 duplication breakpoints in 15 fetuses. Case 1, 2, 3, 8 have smaller typical duplicatons encompassed interval II, while Case 7, 15 have larger atypical duplicatons encompassed interval II and III. The breakpoints of case 4, 5 ,6, 9, 10, 13 encompassed interval I and II, and the breakpoints of case 11, 12, 14 encompassed interval I, II and III. Note: The red rectangle in the chromosome diagram marks the location of 16p13.11. The blue bars indicate the location of duplications in the 16p13.11 region for 15 fetuses. The black bars, from left to right, represent intervals I, II and III respectively. OMIM genes are also labeled, dark green indicates clinical relevant OMIM Morbid genes, and gray indicates other OMIM genes.

SNP array analysis identified duplications in the 16p13.11 region in 15 fetuses (Table 1). The size of fragments ranged from 0.79 to 2.82 Mb, involved 7 to 13 OMIM genes, five of which were morbid genes of clinical relevant diseases (Table 2), The duplications either overlapped or flanked the typical 1.65 Mb 16p13.11 microduplication (chr16:14986684-1648668, GRCh37). These duplications spanned different regions within the single-copy sequence interval ((I, II, III). Out of 15 cases, 9 exhibited typical duplications covering intervals I and II, while cases 7 and 15 showed atypical duplications encompassing intervals II and III. Notably, Interval II was present in all cases (Figure 1).

**TABLE 1:** SNP array of 15 fetuses with 16p13.11 microduplication.

Case	Age (y)	Gestational age (w/d)	SNP-array	OMIM gene	Genes encompassed by the CNV	CNV interval	Size (Mb)	Inheritance
1	36	20w+5d	arr [GRCh37]16p13.11 (15481748_16278133)x3	7	MPV17L- > ABCC6	II	0.79	*De novo*
2	25	27w+5d	arr [GRCh37]16p13.11 (15481748_16309046)x3	7	MPV17L- > ABCC6	II	0.82	Unknown
3	21	21w+6d	arr [GRCh37]16p13.11 (15481748_16309046)x3	7	MPV17L- > ABCC6	II	0.82	Maternal
4	29	19w+3d	arr [GRCh37] 16p13.11 (14892976_16538596)x3	13	NOMO1- > NOMO3	I, II	1.64	Unknown
5	31	20w+1d	arr [GRCh37] 16p13.11 (15129894_16458424)x3	11	PDXDC1- > NOMO3	I, II	1.32	Unknown
6	32	26w+5d	arr [GRCh37] 16p13.11 (14920865_16538596)x3	13	NOMO1- > NOMO3	I, II	1.61	Maternal
7	37	19w	arr [GRCh37] 16p13.11p12.3 (15531257_18151677)x3	8	MARF1- > XYLT1	II,III	2.62	Maternal
8	32	19w+5d	arr [GRCh37] 16p13.11 (15481748_16309046)x3	7	MPV17L- > ABCC6	II	0.82	Maternal
9	38	20w+4d	arr [GRCh37] 16p13.11 (14900043_16508123)x3	13	NOMO1- > NOMO3	I, II	1.60	Unknown
10	42	18w	arr [GRCh37] 16p13.11 (14892976_16508123)x3	13	NOMO1- > NOMO3	I, II	1.61	Paternal
11	25	21w+1d	arr [GRCh37] 16p13.11p12.3 (14892976_16858332)x3	13	NOMO1- > NOMO3	I, II,III	1.96	Maternal
12	22	19w	arr [GRCh37] 16p13.11p12.3 (14892976_16926947)x3	13	NOMO1- > NOMO3	I, II,III	2.03	Unknown
13	30	24w+4d	arr [GRCh37] 16p13.11 (15154357_16538596)x3	10	PDXDC1- > NOMO3	I, II	1.38	Unknown
14	21	20w+1d	arr [GRCh37] 16p13.11p12.3 (15058821_16946747)x3	11	PDXDC1- > NOMO3	I, II,III	1.88	Unknown
15	37	19w+5d	arr [GRCh37] 16p13.11p12.3 (15325073_18151677)x3	9	MPV17L- > XYLT1	II,III	2.82	Unknown

Genomic coordinates (GRCh37) of 3 intervals: I, chr16:15124767-15156253; II, chr16:15511710-16292267; III, chr16:16856795-18167401; y, year; w/d, week/day.

**TABLE 2:** Clinical relevant OMIM Morbid genes of 15 fetuses with 16p13.11 microduplication.

Morbid genes	Cytogenetic location	Genomic coordinates (GRCh37)	Phenotype	Inheritance
*NDE1* (609449)	16p13.11	chr16:15737239-15820210	Lissencephaly 4 (with microcephaly)	AR
			Microhydranencephaly	AR
*MYH11*(160745)	16p13.11	chr16:15796992-15950885	Aortic aneurysm, familial thoracic 4	AD
			Megacystis-microcolon-intestinal hypoperistalsis syndrome 2	AR
			Visceral myopathy 2	AD
*ABCC1*(158343)	16p13.11	chr16: 16043473- 16236910	Deafness, autosomal dominant 77	AD
*ABCC6*(603234)	16p13.11	chr16:16243422-16317351	Arterial calcification, generalized, of infancy, 2	AR
			Pseudoxanthoma elasticum	AR
			Pseudoxanthoma elasticum, forme fruste	AD
*XYLT1* (608124)	16p12.3	chr16:17195626-17564817	Pseudoxanthoma elasticum, modifier of severity of	AR
			Desbuquois dysplasia 2	AR

AR, autosomal recessive; AD, autosomal dominant.

### 3.2 Pathogenicity analysis

The copy number variations (CNVs) in the 16p13.11 region of 15 fetuses were analyzed using public databases. No similar variation reports were included in the DGV database. The DECIPHER database contains multiple cases classified as pathogenic and likely pathogenic, as well as reports of similar variants whose clinical significance remains unclear. According to the ClinGen database, the duplication covers nearly the entire area of the 16p13.11 recurrent region (BP2-BP3, includes MYH11), with a triplosensitivity score of 2, indicating some evidence of pathogenicity. Previous literatures have identified this region as a susceptibility locus for neurocognitive disorders. Duplications in this region have been associated with autism, intellectual developmental delay, and various congenital developmental abnormalities (Ullmann et al., 2007; Mefford et al., 2009). Clinical presentations are generally nonspecific such as developmental delay, intellectual disability, learning difficulties, though asymptomatic carriers have also been reported (Kendall et al., 2019; Allach El Khattabi et al., 2020). The ClinVar database also reports pathogenic and uncertain variants associated with duplications in this region. Based on the ACMG Variant Classification Guidelines (2019), pathogenicity scores ranged from −0.89 to 0.89 points indicating that the clinical significance of these duplications remains uncertain.

### 3.3 Results of SNP-array pedigree analysis

Unfortunately, the parents of 8 fetuses declined pedigree verification. In the remaining 7 fetuses that underwent family validation, one case was *de novo* (1/7), and the remaining 6 cases were inherited from their parents (6/7). Of these, 5 were maternally inherited (5/7), one case was paternally inherited (1/7) with all parents being asymptomatic carriers (Table 1).

### 3.4 Prenatal diagnosis indications

Among the prenatal diagnosis indications for fetuses carrying 16p13.11 microduplication, the most common was ultrasound abnormalities which accounted for 6 of the 15 cases. These abnormalities included structural anomalies (3/15) and soft marker anomalies/abnormal ultrasonic soft indicators (3/15). Structural abnormalities included 1 case of ventricular septal defect, 1 case of omphalocele and 1 case of right kidney agenesis. The soft marker anomalies included 1 case of bilateral choroid plexus cysts combined with a single umbilical artery, 1 case of single umbilical artery and 1 case of unilateral choroid plexus cyst combined with advanced maternal age. NIPT suggested the presence of chromosomal abnormalities in 5 of the 15 cases, with 3 cases showing microduplication of chromosome 16 and 2 cases indicating other chromosomal abnormalities. It is worth noting that advanced maternal age, as a significant indication for prenatal diagnosis, account for 5 of the 15 fetuses carrying 16p13.11 microduplications (Table 3).

**TABLE 3:** Clinical information of 15 fetuses with 16p13.11 microduplication.

Case	Indications for prenatal diagnosis	Pregnancy outcome	Fetal Gender (F/M)	Follow-up age (y/m)	Outcome of live-born
1	Advanced maternal age, microduplication of chromosome 13 for NIPT	Eutocia	F	5 years	Well survivor
2	Ventricular septal defect for ultrasound	Cesarean	M	5 years	Well survivor, Surgery is required due to atrial septal defect and it is easy to catch a cold
3	Microduplication of chromosome 16 for NIPT	Cesarean	M	5 years	Well survivor
4	Balanced translocation of chromosomes in the father of the fetus	Cesarean	M	5 years	Well survivor
5	Bilateral choroid plexus cysts and single umbilical artery for ultrasound	Cesarean	F	5 years	Well survivor
6	single umbilical artery for ultrasound	TP	M	—	—
7	Advanced maternal age, unilateral choroid plexus cyst for ultrasound	Cesarean	F	4 years	Well survivor
8	Reproductive history of children with mental retardation	TP	F	—	—
9	Advanced maternal age, microdeletion of chromosome 10 for NIPT	Cesarean	F	1 year	Well survivor
10	Advanced maternal age	Intrauterine fetal death	M	—	—
11	Microduplication of chromosome 16 for NIPT	Eutocia	M	1 year	Well survivor
12	Reproductive history of children with Down’s syndrome	Cesarean	M	8 months	Well survivor
13	Fetal omphalocele for Ultrasound	Eutocia	F	8 months	Well survivor, umbilical hernia, no surgery currently required
14	Fetal right kidney absence for Ultrasound	Lost to follow-up	F	—	—
15	Microduplication of chromosome 16 for NIPT	Eutocia	F	4 months	Well survivor

NIPT, non-invasive prenatal testing; TP, termination of pregnancy; F/M, female/male; y/m, year/month.

### 3.5 Pregnancy outcome

Of the 15 fetuses with 16p13.11 microduplications, except for one fetus was lost to follow-up, the pregnancies of an additional 3 fetuses were terminated. The remaining 11 fetuses carried to term following thorough adequate genetic counseling regarding the associated risks (Table 3). The age of the 11 surviving children ranged from 4 months to 5 years till the time of publication. One child with a ventricular septal defect was last followed up at 5 years of age. The child displayed normal intelligence, motor skills and language, which were confirmed by his patents. But they noted a susceptibility to colds, as the child had not undergone heart surgery. Another child with omphalocele was last followed up at 8 months and was reported to be able to roll over and sit independently, with a present lack of need for surgery. The other 9 children exhibited no notable abnormalities based on information come from their parents.

## 4 Discussion

The short arm of chromosome 16 (16p) is a hotspot region for chromosomal copy number variations (CNVs), particularly the 16p13.11 region (Ciaccio et al., 2017; Redaelli et al., 2019; Atli et al., 2022). This region consists of three single-copy sequence intervals (I, II, III), flanked by low copy repeats (LCRs) (Ingason et al., 2011), which predispose to chromosomal rearrangements through non-allelic homologous recombination (NAHR). Intervals I and II are critical for copy number variation (Nagamani et al., 2011). In this study, SNP array analysis of amniotic fluid of 3,451 pregnant women revealing 15 fetuses with varying sizes of microduplications in the 16p13.11 region, ranging from 0.79 to 2.82 Mb. The breakpoints of 15 cases with 16p13.11 microduplications covered different regions across the three single-copy sequence intervals (I, II, III). Notably, There were 9 cases whose breakpoints encompassed intervals I and II, and all cases involved interval II. The concentration of breakpoints in intervals I and II suggests that these two regions may harbor key genes associated with clinical phenotype, consistent with previous findings (Nagamani et al., 2011). Interval I located between 15.12 and 15.15 Mb, contains 3 OMIM genes, including *NTAN1*, which encodes an asparagine-specific N-terminal amidase, which is an enzyme involved in regulating the half-life of proteins *in vivo*. Inactivation of the *Ntan1* in mice is associated with abnormal neural activities, such as changes in social behavior, impaired memory, impairment of spatial and non-spatial learning abilities (Kwon et al., 2000; Balogh et al., 2001). Interval II located between 15.51 and 16.29 Mb, contains 7 OMIM genes, including *NDE1*, which is highly expressed in the brain and plays a pivotal role in the growth and development of cerebral cortex (Luttik et al., 1998; Nagamani et al., 2011). Knockout of *Nde1* in mice leads to abnormalities in the cerebral cortex and microcephaly (Feng and Walsh, 2004). Moreover, *NDE1* gene is regarded as a strong candidate gene associated with phenotypes of neural development (Soto-Perez et al., 2020). Interval II identified as a dosage-sensitive region, represents the core pathogenic locus of 16p13.11 (Tropeano et al., 2013), which consistents with our findings that all cases encompassed interval II.

The 16p13.11 microduplication is recognized as a neurodevelopmental susceptibility locus with incomplete penetrance and variable expression, which is an important impact on developmental delay, learning difficulties and behavioral abnormalities (Coe et al., 2014; Nuttle et al., 2016). The phenotype in adults and children has been well described in the previous literatures, such as cognitive impairment, behavioral abnormalities, cardiac and aortic malformations, skeletal abnormalities and other organ abnormalities (Balogh et al., 2001; Hamad et al., 2023).

However, there are few reports on the prenatal phenotype associated with this microduplication. Redaelli (Redaelli et al., 2019) detected 366 prenatal specimens using comparative genomic hybridization (aCGH) and found that one fetus with prenatal indications of non-immune edema carried 16p13.11 microduplications. Dabkowska (Dabkowska et al., 2020) identified a 16p13.11 microduplication in a fetus with encephalocele. Cai (Cai et al., 2022) found 15 fetuses with 16p13.11 microduplication by SNP array detected from prenatal diagnosis of 9,000 pregnant women, which indicated the most relevant prenatal indicators were structural or soft marker anomalies on ultrasound. Similarly, in this study, ultrasound abnormalities were the most common indication for prenatal diagnosis observed in 6 out of 15 cases, including 3 with structural abnormalities and 3 with soft marker anomalies. Hamad (Hamad et al., 2023) noted congenital cardiac anomalies in 16 of 206 patients with 16p13.11 microduplication, with the most common feature of which was ventricular septal defect. Houcinat (Houcinat et al., 2015) described a family with congenital renal and urinary system anomalies associated with 16p13.11 microduplication, in which the father had unilateral kidney dysplasia and the son suffered from chronic kidney disease and pelvic ureteral obstruction. Shi (Shi et al., 2021) reported that the rate of chromosomal abnormalities was as high as 29.6% on fetal omphalocele. These findings highlight the association between ultrasound abnormalities or soft marker anomalies and chromosomal rearrangements involving 16p13.11 region (Lan et al., 2020; Su et al., 2021). In our cohort, NIPT suggested chromosomal abnormalities in 5 out of 15 cases, 3 of which involved microduplication of chromosome 16. With the widespread clinical application of NIPT, the detection rate of CNVs involving 16p13.11 microduplications has been greatly improved (Du et al., 2018). Advanced maternal age, a known high-risk factor for chromosomal abnormalities (Wang et al., 2019), was the prenatal diagnosis indication in 5 out of 15 cases carrying 16p13.11 microduplications, possibly contributing to the higher detection rate in our study. The specific relationship between advanced age factors and the occurrence of 16p13.11 microduplications is still unclear.

Initially speculated a “benign variant” due to its presence in phenotypically normal parents, however, the pathogenicity of 16p13.11 microduplication is gradually recognized as more patients detected. (Hannes et al., 2009; Nagamani et al., 2011). The penetrance is estimated at around 7%–10.6% (Kendall et al., 2019; Kirov et al., 2014), and the duplication may be inherited from asymptomatic parents or occur *de novo* (Redaelli et al., 2019). It has been reported that approximately 93.6% of CNVs inherited from unaffected parents (Nagamani et al., 2011; Hamad et al., 2023). In our study, 7 cases underwent pedigree verification, and one case was *de novo* (1/7), while the remaining 6 were inherited (6/7), accounting for about 85.7% inherited from asymptomatic parents, consistent with previous literature. Notably, 5 cases were inherited from the mother (5/7), while one case had paternal inheritance (1/7), indicating a higher maternal inheritance rate. Tropeano (Tropeano et al., 2013) found a male bias in the 16p13.11 region, but there was no gender bias in our samples (7 males and 8 females), likely due to the small sample size.

Our follow-up revealed a 15.4% penetrance rate via telephone follow-up, which is higher than the levels reported in the literatures (Kendall et al., 2019; Kirov et al., 2014; Martin et al., 2020). There are two children exhibiting clinical phenotypes: one with ventricular septal defect and the other with omphalocele. This discrepancy may be affected by the limited sample size and the nature of telephone follow-up of our study, which may influence parental reports Additionally, genetic factors beyond 16p13.11 CNVs, such as other undetected mutations, could contribute to the phenotypes, but further testing was declined by their parents. Children with 16p13.11 microduplications might lack of specific phenotypes and the abnormalities may progressively emerge during growth or relatively mild. Although no abnormalities are evident in the neonatal period, affected individuals may show varying degrees of cognitive impairments and behavioral abnormalities with increasing age (Delicado et al., 2014). Therefore, ongoing monitoring of the growth and development of individuals with 16p13.11 microduplications is crucial.

In conclusion, when CNVs in the 16p13.11 region are detected during fetal development, clinical decisions should be based on a comprehensive analysis of fetal ultrasound findings, familial history and detailed genetic counseling instead of terminating the pregnancy solely. The potential for varying phenotypic expression within the same family underscores the importance of considering the origin in the parental generation and recurrence risks in comprehensive genetic counseling.

This study has several limitations. First, the sample size was small, with only 15 cases of fetal 16p13.11 microduplications detected. Second, the point mutations of genes were not detected by SNP array used in this study, but the next-generation sequencing was not employed. Third, the longest follow-up case in our study was tracked up to 5 years and clinical phenotypes associated with 16p13.11 microduplications are mainly observed in adults and children. So it is essential to conduct long-term follow-ups of these children in future studies to observe their growth and development. In future, research with larger sample size and longer follow-up period are necessary to better understand the genotype-phenotype correlation and provide more comprehensive genetic counseling.

## 5 Conclusion

The use of SNP array is an effective tool for diagnose variations in the 16p13.11 region. Our findings indicate that microduplication of 16p13.11 is strongly associated structural and soft marker anomalies detected via prenatal ultrasound. The application of NIPT (non-invasive prenatal testing) has enhanced the detection rate of prenatal 16p13.1 microduplications. Simultaneously, comprehensive prenatal phenotypic analyses, follow-ups on assessments of pregnancy outcomes and postnatal follow-ups for fetuses with 16p13.11 microduplications were conducted in this study. The majority of these fetuses were found to be in good health after birth, offering valuable insights for clinical genetic counseling.

## Data Availability

The datasets presented in this study can be found in online repositories. The names of the repository/repositories and accession number(s) can be found in the article/supplementary material.
